# Rural-Urban Disparities in Hepatocellular Carcinoma Incidence and Mortality

**DOI:** 10.1001/jamanetworkopen.2026.12323

**Published:** 2026-05-13

**Authors:** Haluk Damgacioglu, Gokhan Uygun, Ashish A. Deshmukh, Ahmed O. Kaseb, Anne M. Noonan, Tarik Demir

**Affiliations:** 1 Department of Public Health Sciences, Medical University of South Carolina, Charleston; 2 Cancer Control Program, Hollings Cancer Center, Medical University of South Carolina, Charleston; 3 Division of Medical Oncology, Department of Internal Medicine, Canakkale Onsekiz Mart University Faculty of Medicine, Canakkale, Turkey; 4 Department of Gastrointestinal Medical Oncology, The University of Texas MD Anderson Cancer Center, Houston; 5 Division of Medical Oncology, Department of Internal Medicine, The Ohio State University Comprehensive Cancer Center, Columbus

## Abstract

**Importance:**

Although hepatocellular carcinoma (HCC) incidence has decreased in the US, it is unclear if rural residents have experienced similar trends.

**Objective:**

To examine rural-urban differences in HCC incidence and incidence-based mortality trends by sex, race and ethnicity, and stage at diagnosis.

**Design, Setting, and Participants:**

This cohort study analyzed HCC diagnoses from 2001 to 2022 and deaths from 2007 to 2022 using data from the National Program of Cancer Registries and the Surveillance, Epidemiology, and End Results (SEER) programs for incidence and the SEER-21 program for incidence-based mortality. Rurality was classified using the 2013 Rural-Urban Continuum Codes. All analyses were performed from October 2025 to February 2026.

**Main Outcomes and Measures:**

Annual incidence and incidence-based mortality rates per 100 000 people were calculated and age standardized to the 2000 US standard population. The annual percentage change (APC) and the average APC in incidence and incidence-based mortality rates were then estimated using Joinpoint regression.

**Results:**

The study analyzed 264 633 HCC cases (77.0% among men and 86.6% in urban areas). Among men, HCC incidence was 4.5 (95% CI, 4.4-4.5) per 100 000 people in rural counties and 5.8 (95% CI, 5.8-5.8) per 100 000 people in urban counties; among women, HCC incidence was 1.2 (95% CI, 1.2-1.2) per 100 000 people in rural counties and 1.5 (95% CI, 1.5-1.5) per 100 000 people in urban counties. In rural counties, HCC incidence increased by 1.1% (95% CI, 0.6%-1.6%) per year among men from 2007 to 2022 and 1.7% (95% CI, 1.2%-2.3%) per year among women from 2001 to 2022, whereas in urban counties, HCC rates decreased by 1.4% (95% CI, −1.7% to −1.1%) per year among men from 2008 to 2022 and by 1.0% (95% CI, −1.4% to −0.6%) per year among women from 2009 to 2022. Among men, incidence-based mortality rates were 2.7 (95% CI, 2.3-3.0) per 100 000 people in rural counties and 3.8 (95% CI, 3.6-3.9) per 100 000 people in urban counties; among women, the incidence-based mortality rates were 0.8 (95% CI, 0.6-0.9) per 100 000 people in rural counties and 0.9 (95% CI, 0.8-1.0) per 100 000 people in urban counties. In rural counties, incidence-based mortality rates increased by 1.2% (95% CI, 0.3%-2.1%) per year among men and remained stable among women (APC, 0.3% [95% CI, −1.1% to 1.7%]), whereas in urban counties, incidence-based mortality rates decreased by 1.4% per year (95% CI, −1.7% to −1.1%) among men and 1.0% (95% CI, −1.7% to −0.4%) per year among women.

**Conclusions and Relevance:**

This cohort study revealed rural-urban disparities in HCC incidence and incidence-based mortality trends. These findings highlight the need for targeted prevention strategies and improved access to early detection and specialty care for rural populations.

## Key Points

**Question:**

Are there disparities in hepatocellular carcinoma (HCC) incidence and mortality between rural and urban populations in the US?

**Findings:**

In this cohort study, population-based analysis from 2001 to 2022 demonstrated stable or increased HCC incidence and mortality rates in rural counties and decreased rates in urban counties. These trends were particularly evident among the non-Hispanic White population.

**Meaning:**

These findings emphasize the growing disparity between rural and urban populations in HCC incidence and mortality trends and highlight the need for enhanced strategies in liver disease prevention, early detection, and health care accessibility in rural regions.

## Teaser

This cohort study examines rural-urban disparities from 2001 to 2022 in hepatocellular carcinoma incidence and mortality trends by sex, race and ethnicity, and stage at diagnosis.

## Introduction

Hepatocellular carcinoma (HCC) is the most common primary liver cancer, accounting for approximately 90% of liver cancers.^1^ Major risk factors for HCC include chronic viral hepatitis, alcohol-related liver disease (ALD), aflatoxin exposure, obesity, and metabolic dysfunction–associated steatohepatitis (MASH). In the US, HCC incidence increased steadily from the 1970s through the early 2010s, driven primarily by chronic viral hepatitis infections.^2^ Recent studies indicate that HCC incidence has begun to decrease among several racial and ethnic groups, reflecting improvements in viral hepatitis treatment and prevention.^3^ However, emerging evidence suggests a shifting etiology of HCC, with MASH, obesity, diabetes, and ALD becoming increasingly important factors associated with the disease.^4,5^

These risk factors are more common in rural populations, raising concerns about geographic disparities in HCC burden.^4,5^ Despite the documented national decreases in HCC incidence, it remains unclear whether rural populations have experienced similar improvements.^6^ Therefore, in this study, we aimed to examine rural-urban disparities in HCC incidence and incidence-based mortality trends in the US from 2001 to 2022. We further evaluated trends by sex, race and ethnicity, and stage at diagnosis to better understand geographic differences in HCC incidence and mortality patterns.

## Methods

### Data Sources

This study used data from the National Program of Cancer Registries (NPCR) and Surveillance, Epidemiology, and End Results (SEER) databases. For the analysis of incidence-based mortality, which measures liver cancer mortality among individuals with a diagnosis of HCC, we used the SEER-21 registry incidence-based mortality file, which links SEER-21 cancer incidence data with death certificate information.^5^ We identified HCC cases using site code C22.0 and histology codes 8170 to 8175, based on the *International Classification of Diseases for Oncology, Third Edition*.^7^ This cohort study followed the Strengthening the Reporting of Observational Studies in Epidemiology (STROBE) reporting guideline for cohort studies and was deemed exempt by the Medical University of South Carolina institutional review board, as the data were deidentified and publicly available, in accordance with 45 CFR §46.

### Statistical Analysis

Statistical analysis was conducted from October 2025 to February 2026. Using SEER*Stat, version 9.0.41.4 (National Cancer Institute), we estimated annual incidence and incidence-based mortality rates per 100 000 people, age adjusted to the 2000 US standard population. To prevent underestimation of incidence-based mortality rates in earlier years due to limited follow-up time, we included diagnoses from 2000 to 2022 and deaths from 2007 to 2022. Rurality was assessed using the 2013 Rural-Urban Continuum Codes, with codes 1 to 3 indicating urban areas and codes 4 to 9 indicating rural areas. Incidence and incidence-based mortality rates were further stratified by sex and race and ethnicity. Race and ethnicity were obtained from the NPCR-SEER database and classified as Hispanic, non-Hispanic Black, non-Hispanic White, and non-Hispanic other, which included non-Hispanic American Indian or Alaska Native, non-Hispanic Asian or Pacific Islander, and unknown. Due to data suppression for the non-Hispanic Asian or Pacific Islander population, particularly in rural areas, we combined the non-Hispanic American Indian or Alaska Native and Asian or Pacific Islander populations and reported them as non-Hispanic other. Race and ethnicity data were collected to assess potential differences in HCC incidence and mortality across demographic groups. We further analyzed incidence by stage at diagnosis using the summary stage variable in the database, which categorizes cases as localized, regional, distant, or unknown or unstaged.

We estimated piecewise log-linear trends in incidence and calculated annual percentage changes (APCs) with 95% CIs using the Joinpoint regression program, version 5.4.0 (National Cancer Institute). We excluded 2020 from the HCC incidence trend analysis, as the COVID-19 pandemic may have affected incidence reporting.

## Results

**Figure 1.**
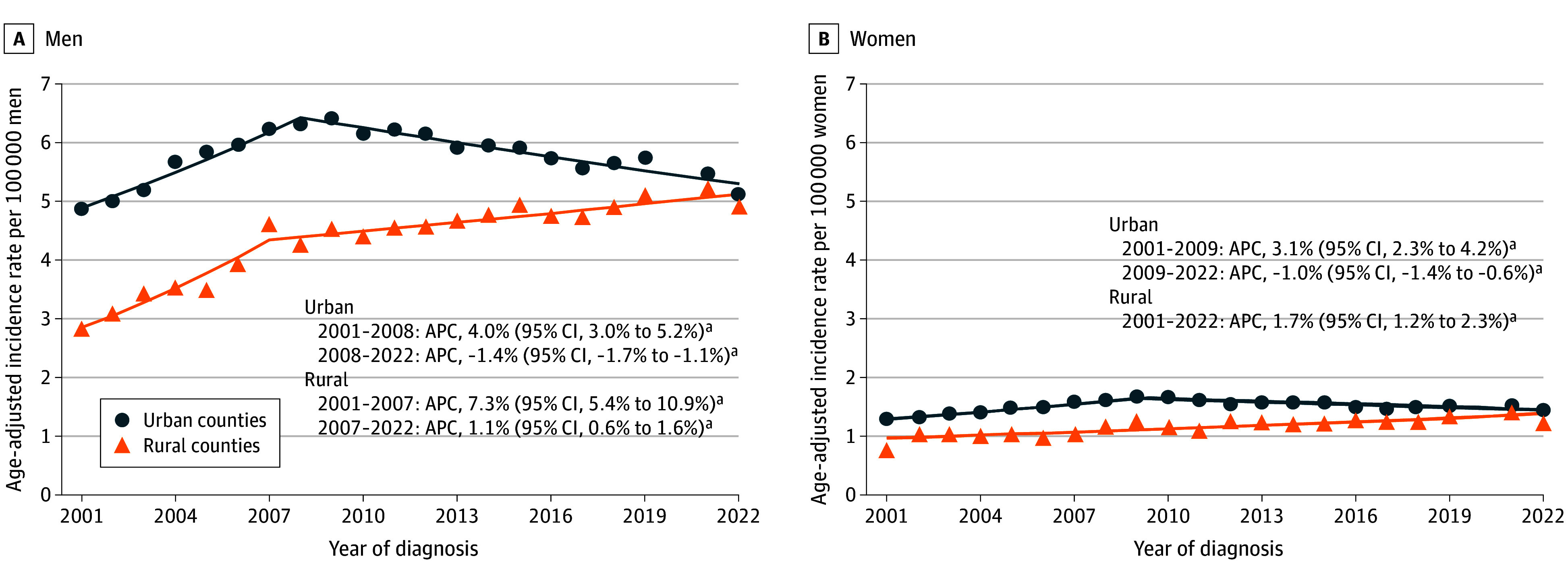
Scatterplots Showing Age-Adjusted Incidence of Hepatocellular Carcinoma by Sex and Rurality in the US, 2001-2022 The scatterplots display annual age-adjusted incidence rates of hepatocellular carcinoma in urban and rural counties between 2001 and 2022. Solid lines indicate modeled incidence trends estimated using Joinpoint regression. Hepatocellular carcinoma cases were identified using the *International Classification of Diseases for Oncology, Third Edition* (site code C22.0; histology codes 8170-8175). Rurality was determined using the 2013 Rural-Urban Continuum Codes and categorized as urban counties (codes 1-3) and rural counties (codes 4-9). Incidence rates were age adjusted to the 2000 US standard population and expressed per 100 000 persons. Annual percentage changes (APCs) and 95% CIs were estimated using the Joinpoint Regression Program, version 5.4.0 (National Cancer Institute). Calendar segments correspond to statistically identified joinpoints. ^a^*P* < .05.

From 2001 to 2022, a total of 264 633 people (77.0% among men and 23.0% among women; 86.6% in urban areas; 14.8% Hispanic, 14.5% non-Hispanic Black, 61.2% non-Hispanic White, and 9.5% non-Hispanic other) received a diagnosis of HCC. Among men, HCC incidence rates were 4.5 (95% CI, 4.4-4.5) per 100 000 people in rural counties and 5.8 (95% CI, 5.8-5.8) per 100 000 people in urban counties. Among men in rural counties, the incidence increased by 1.1% (95% CI, 0.6%-1.6%) per year from 2007 to 2022, after an earlier increase of 7.3% (95% CI, 5.4%-10.9%) per year from 2001 to 2007 (Table and Figure 1). In contrast, men in urban counties experienced a decrease in incidence from 2008 to 2022 (APC, −1.4% [95% CI, −1.7% to −1.1%]) after a prior increase from 2001 to 2008 (APC, 4.0% [95% CI, 3.0%-5.2%]). For women, the HCC incidence was 1.2 (95% CI, 1.2-1.2) per 100 000 people in rural counties and 1.5 (95% CI, 1.5-1.5) per 100 000 people in urban counties. Among women in rural counties, incidence increased by 1.7% (95% CI, 1.2%-2.3%) per year from 2001 to 2022, whereas among women in urban counties, the incidence increased by 3.1% (95% CI, 2.3%-4.2%) per year from 2001 to 2009 and then decreased by 1.0% (95% CI, −1.4% to −0.6%) per year from 2009 to 2022.

**Table.** Trends in Hepatocellular Carcinoma Incidence, Stage at Diagnosis–Specific Incidence (2001-2022), and Incidence-Based Mortality (2007-2022) by Race and Ethnicity, Sex, and Rurality in the US^a^

Population^c^	Urban counties^b^					Rural counties^b^				
	Years	Rate per 100 000		Annual percentage change, % (95% CI)^d^	Average annual percentage change, % (95% CI)^d^	Years	Rate per 100 000		Annual percentage change, % (95% CI)^d^	Average annual percentage change, % (95% CI)^d^
		First year	Final year				First year	Final year		
**Incidence by race and ethnicity, stratified by sex** ^e^										
Men										
Overall	2001-2008	4.9	6.4	4.0 (3.0 to 5.2)^f^	0.4 (0.2 to 0.6)^f^	2001-2007	2.9	4.4	7.3 (5.4 to 10.9)^f^	2.8 (2.5 to 3.4)^f^
	2008-2022	6.4	5.3	−1.4 (−1.7 to −1.1)^f^		2007-2022	4.4	5.1	1.1 (0.6 to 1.6)^f^	
Hispanic	2001-2006	9.1	10.5	3.9 (1.3 to 9.2)^f^	−0.8 (−1.2 to −0.2)^f^	2001-2022	7.1	9.3	0.8 (0.0 to 1.8)	0.8 (0.0 to 1.8)
	2006-2022	10.5	6.9	−2.2 (−2.7 to −1.8)^f^						
Non-Hispanic Black	2001-2008	6.8	9.6	5.5 (3.8 to 8.3)^f^	0.0 (−0.4 to 0.5)	2001-2009	3.3	7.0	6.2 (2.7 to 29.6)^f^	2.4 (0.9 to 4.8)^f^
	2008-2022	9.6	6.7	−2.7 (−3.3 to −2.1)^f^		2009-2022	7.0	6.3	0.1 (−5.3 to 1.6)	
Non-Hispanic White	2001-2008	3.8	5.0	4.3 (3.3 to 5.7)^f^	0.8 (0.6 to 1.1)^f^	2001-2007	2.6	4.3	7.6 (5.6 to 11.3)^f^	2.9 (2.6 to 3.5)^f^
	2008-2022	5.0	4.3	−0.9 (−1.3 to −0.6)^f^		2007-2022	4.3	4.5	1.1 (0.6 to 1.6)^f^	
Non-Hispanic other	2001-2007	13.5	13.7	−0.4 (−1.8 to 3.5)	−2.6 (−3.0 to −2.2)^f^	2001-2013	8.4	9.7	1.8 (0.3 to 30.9)^f^	0.2 (−1.2 to 2.9)
	2007-2022	13.7	7.6	−3.5 (−4.1 to −3.1)^f^		2013-2022	9.7	7.8	−2.0 (−10.1 to 0.3)	
Women										
Overall	2001-2009	1.2	1.7	3.1 (2.3 to 4.2)^f^	0.5 (0.3 to 0.8)^f^	2001-2022	0.8	1.2	1.7 (1.2 to 2.3)^f^	1.7 (1.2 to 2.3)^f^
	2009-2022	1.7	1.5	−1.0 (−1.4 to −0.6)^f^						
Hispanic	2002-2006	2.9	3.3	2.2 (−0.3 to 10.5)	−0.5 (−1.0 to 0.1)	2002-2022	2.8	2.5	0.2 (−1.4 to 2.1)	0.2 (−1.4 to 2.1)
	2006-2022	3.3	2.6	−1.4 (−2.3 to −1.0)^f^						
Non-Hispanic Black	2002-2010	1.6	2.4	4.1 (2.4 to 7.4)^f^	0.1 (−0.6 to 0.9)	2002-2022	1.3	1.3	0.1 (−1.4 to 1.6)	0.1 (−1.4 to 1.6)
	2010-2022	2.4	1.7	−2.9 (−4.5 to −1.8)^f^						
Non-Hispanic White	2001-2008	1.0	1.2	3.5 (2.2 to 6.1)^f^	1.0 (0.7 to 1.4)^f^	2001-2022	0.7	1.1	1.8 (1.1 to 2.5)^f^	1.8 (1.1 to 2.5)^f^
	2008-2022	1.2	1.1	−0.3 (–0.9 to 0.2)						
Non-Hispanic other	2004-2008	4.1	4.1	−1.3 (−3.8 to 18.1)	−3.3 (−4.5 to −1.8)^f^	2004-2022	5.0	2.1	−2.1 (−3.9 to −0.1)^f^	−2.1 (−3.9 to −0.1)^f^
	2008-2022	4.1	2.2	−4.3 (−9.9 to −3.1)^f^						
**Incidence by stage at diagnosis, stratified by sex** ^e^ ^,^ ^g^										
Men										
Localized	2001-2007	1.9	3.1	8.8 (7.3 to 10.9)^f^	1.5 (1.1 to 1.8)^f^	2001-2007	1.1	2.0	10.8 (7.2 to 19.1)^f^	4.0 (3.3 to 5.2)^f^
	2007-2013	3.1	2.8	–1.8 (–4.3 to –0.7)^f^						
	2013-2019	2.8	2.9	1.0 (0.2 to 3.9)^f^		2007-2022	2.0	2.4	1.4 (0.4 to 2.2)^f^	
	2019-2022	2.9	2.5	–4.9 (–6.9 to –2.4)^f^						
Regional	2001-2006	1.1	1.6	6.4 (3.8 to 16.7)^f^	0.9 (0.3 to 1.7)^f^	2001-2013	0.7	1.4	5.5 (4.5 to 6.7)^f^	2.6 (2.1 to 3.2)
	2006-2014	1.6	1.7	0.9 (–0.8 to 2.5)						
						2013-2018	1.4	1.1	–4.9 (–10.0 to –2.3)^f^	
	2014-2017	1.7	1.2	–10.3 (–13.1 to –5.6)^f^						
						2018-2022	1.1	1.3	4.0 (0.4 to 9.1)^f^	
	2017-2022	1.2	1.4	2.4 (–0.3 to 8.5)						
Distant	2001-2008	0.9	1.1	3.0 (2.0 to 4.6)^f^	0.6 (0.4 to 0.9)^f^	2001-2007	0.5	0.8	7.9 (4.5 to 21.2)	3.6 (2.9 to 4.8)
	2008-2022	1.1	1.0	–0.6 (–1.0 to –0.3)^f^		2007-2022	0.8	1.1	1.9 (0.6 to 2.7)	
Unknown or unstaged	2001-2005	1.0	0.6	−10.8 (−19.5 to −5.6)^f^	−5.5 (−6.4 to −4.7)^f^	2001-2015	0.6	0.4	−3.1 (−13.8 to −0.4)^f^	−3.1 (−5.2 to −1.6)^f^
	2005-2019	0.6	0.5	−2.3 (−3.0 to 2.8)		2015-2019	0.4	0.6	10.1 (−1.5 to 22.2)	
	2019-2022	0.5	0.3	−12.9 (−18.5 to −5.4)^f^		2019-2022	0.6	0.3	−18.0 (−30.4 to −1.3)^f^	
Women										
Localized	2001-2007	0.5	0.9	8.5 (6.7 to 11.0)^f^	2.1 (1.8 to 2.4)^f^	2001-2008	0.4	0.6	7.3 (3.6 to 26.8)^f^	3.1 (1.9 to 5.1)^f^
	2007-2022	0.9	0.8	–0.4 (–0.8 to 0.0)^f^		2008-2022	0.6	0.7	1.1 (–2.5 to 2.3)	
Regional	2001-2013	0.3	0.4	2.6 (1.6 to 4.1)^f^	0.7 (−0.0 to 1.3)	2001-2012	0.2	0.3	4.1 (2.2 to 25.2)^f^	1.9 (0.4 to 4.1)^f^
	2013-2017	0.4	0.3	–8.0 (–13.5 to –3.7)^f^						
						2012-2022	0.3	0.3	–0.6 (–8.4 to 1.6)	
	2017-2022	0.3	0.3	3.2 (–0.7 to 10.7)						
Distant	2001-2022	0.2	0.2	0.1 (–0.2 to 0.6)	0.1 (−0.2 to 0.6)	2001-2022	0.1	0.2	2.0 (0.8 to 3.3)^f^	2.0 (0.8 to 3.3)^f^
Unknown or unstaged	2001-2022	0.2	0.1	−3.4 (−4.5 to −2.3)^f^	−3.4 (−4.5 to −2.3)^f^	2001-2005	0.2	0.1	−14.6 (−29.3 to −5.8)^f^	−3.2 (−4.4 to −1.7)^f^
						2005-2022	0.1	0.1	−0.3 (−1.8 to 2.6)	
**Incidence-based mortality by sex and race and ethnicity** ^e^										
Men										
Overall	2007-2012	3.8	3.9	0.0 (−1.0 to 1.5)	−1.4 (−1.7 to −1.1)^f^	2007-2022	2.7	3.5	1.2 (0.3 to 2.1)^f^	1.2 (0.3 to 2.1)^f^
	2012-2020	3.9	2.9	−3.4 (−4.5 to −3.0)^f^						
	2020-2022	2.9	3.1	3.3 (−0.2 to 5.4)						
Hispanic	2007-2022	5.6	4.5	−2.8 (−3.4 to −2.1)^f^	−2.8 (−3.4 to −2.1)^f^	2007-2022	6.8	7.4	0.6 (−1.8 to 3.4)	0.6 (−1.8 to 3.4)
Non-Hispanic Black	2007-2009	4.9	6.0	10.0 (−1.6 to 21.2)	−2.0 (−3.3 to −0.8)^f^	2007-2017	3.0	5.6	5.4 (−3.0 to 11.3)	2.5 (−0.4 to 4.6)
						2017-2020	5.6	3.1	−17.8 (−26.2 to 13.4)	
	2009-2022	6.0	3.7	−3.7 (−6.0 to −3.0)^f^						
						2020-2022	3.1	5.0	24.0 (−7.4 to 48.5)	
Non-Hispanic White	2007-2012	2.8	2.9	0.7 (−0.5 to 2.6)	−0.8 (−1.2 to −0.4)^f^	2007-2022	2.3	2.9	1.2 (0.5 to 2.0)^f^	1.2 (0.5 to 2.0)^f^
	2012-2020	2.9	2.3	−3.2 (−4.6 to −2.6)^f^						
	2020-2022	2.3	2.5	5.6 (1.2 to 8.2)^f^						
Non-Hispanic other	2007-2022	7.2	4.0	−4.5 (−5.1 to −3.9)^f^	−4.5 (−5.1 to −3.9)^f^	2007-2022	4.6	4.1	−2.2 (−5.1 to 1.0)	−2.2 (−5.1 to 1.0)
Women										
Overall	2007-2010	0.9	1.0	3.6 (0.0 to 10.2)	−1.0 (−1.7 to −0.4)^f^	2007-2022	0.8	0.8	0.3 (−1.1 to 1.7)	0.3 (−1.1 to 1.7)
	2010-2020	1.0	0.8	−3.4 (−5.8 to −2.9)^f^						
	2020-2022	0.8	0.9	4.4 (−1.7 to 7.9)						
Hispanic	2007-2022	1.7	1.3	−2.6 (−3.8 to −1.3)^f^	−2.6 (−3.8 to −1.3)^f^	2007-2022	2.1	1.3	−1.1 (−5.6 to 4.1)	−1.1 (−5.6 to 4.1)
Non-Hispanic Black	2007-2011	1.0	1.6	9.7 (6.0 to 17.0)^f^	−1.4 (−2.1 to −0.2)^f^	2007-2022	1.0	0.1	−2.0 (−6.8 to 3.0)	−2.0 (−6.8 to 3.0)
	2011-2014	1.6	1.0	−10.7 (−13.8 to −5.2)^f^						
	2014-2022	1.0	0.8	−2.9 (−4.4 to 3.2)						
Non-Hispanic White	2007-2022	0.6	0.6	−1.7 (−2.7 to −0.8)^f^	−1.7 (−2.7 to −0.8)^f^	2007-2022	0.6	0.7	0.6 (−0.7 to 2.0)	0.6 (−0.7 to 2.0)
Non-Hispanic other	2007-2010	1.7	2.2	7.1 (−2.8 to 29.5)	−2.6 (−4.5 to −0.7)^f^	2007-2022	2.6	1.7	−2.0 (−5.9 to 2.5)	−2.0 (−5.9 to 2.5)
	2010-2020	2.2	0.8	−8.2 (−18.2 to −6.8)^f^						
	2020-2022	0.8	1.1	13.1 (−4.7 to 25.7)						

^a^ Hepatocellular carcinoma cases were identified using the *International Classification of Diseases for Oncology, Third Edition *(site code C22.0; histology codes 8170-8175). Incidence-based mortality included liver cancer deaths among individuals who previously received a diagnosis of hepatocellular carcinoma, based on the Surveillance, Epidemiology, and End Results (SEER)–21 incidence-based mortality file, which links SEER-21 incidence data with death certificate data. Race and ethnicity were categorized as Hispanic, non-Hispanic Black, non-Hispanic White, and non-Hispanic other (American Indian or Alaska Native, Native Hawaiian or Pacific Islander, and unspecified race categories).

^b^ Rurality was assessed using 2013 Rural-Urban Continuum Codes and categorized as urban (codes 1-3) or rural (codes 4-9).

^c^ For some subgroups, trend analyses began after 2001 due to fewer than 16 cases in earlier years, consistent with SEER data reporting standards.

^d^ Estimated using Joinpoint Regression Program, version 5.4.0 (National Cancer Institute).

^e^ Rates were age adjusted to the 2000 US standard population and expressed per 100 000 persons.

^f^ Statistically significant results (*P* < .05).

^g^ Stages were categorized as localized, regional, distant, and unknown or unstaged.

**Figure 2.**
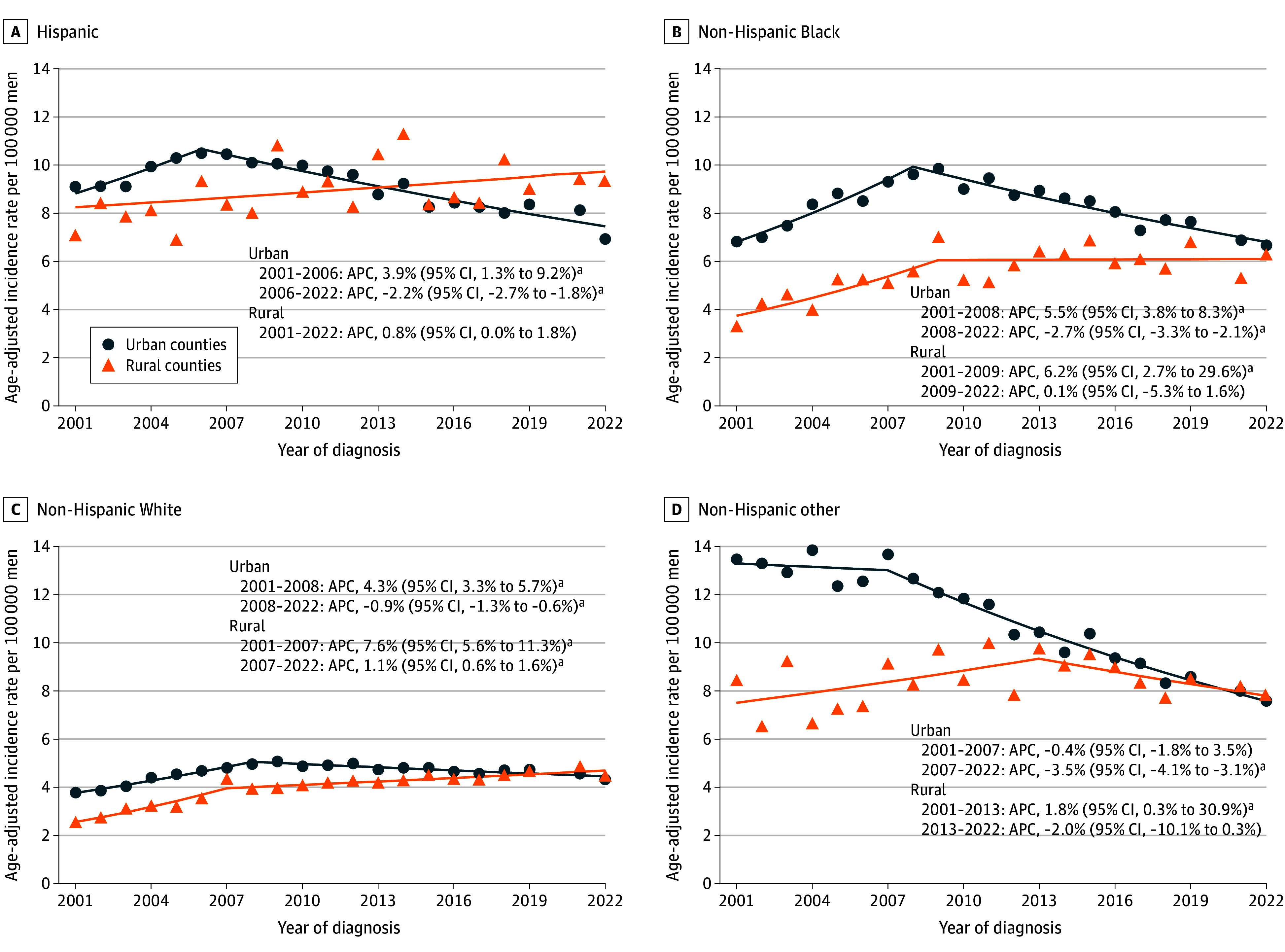
Scatterplots Showing Age-Adjusted Incidence of Hepatocellular Carcinoma Among Men in the US by Race and Ethnicity and Rurality, 2001-2022 The scatterplots display annual age-adjusted incidence rates of hepatocellular carcinoma in urban and rural counties between 2001 and 2022. Race and ethnicity were categorized as Hispanic, non-Hispanic Black, non-Hispanic White, and non-Hispanic other (American Indian or Alaska Native, Native Hawaiian or Pacific Islander, and unspecified race categories). Hepatocellular carcinoma cases were identified using the *International Classification of Diseases for Oncology, Third Edition* (site code C22.0; histology codes 8170-8175). Rurality was determined using the 2013 Rural-Urban Continuum Codes and categorized as urban counties (codes 1-3) and rural counties (codes 4-9). Incidence rates were age adjusted to the 2000 US standard population and expressed per 100 000 persons. Annual percentage changes (APCs) and 95% CIs were estimated using the Joinpoint Regression Program, version 5.4.0 (National Cancer Institute). Calendar segments correspond to statistically identified joinpoints. ^a^*P* < .05.

**Figure 3.**
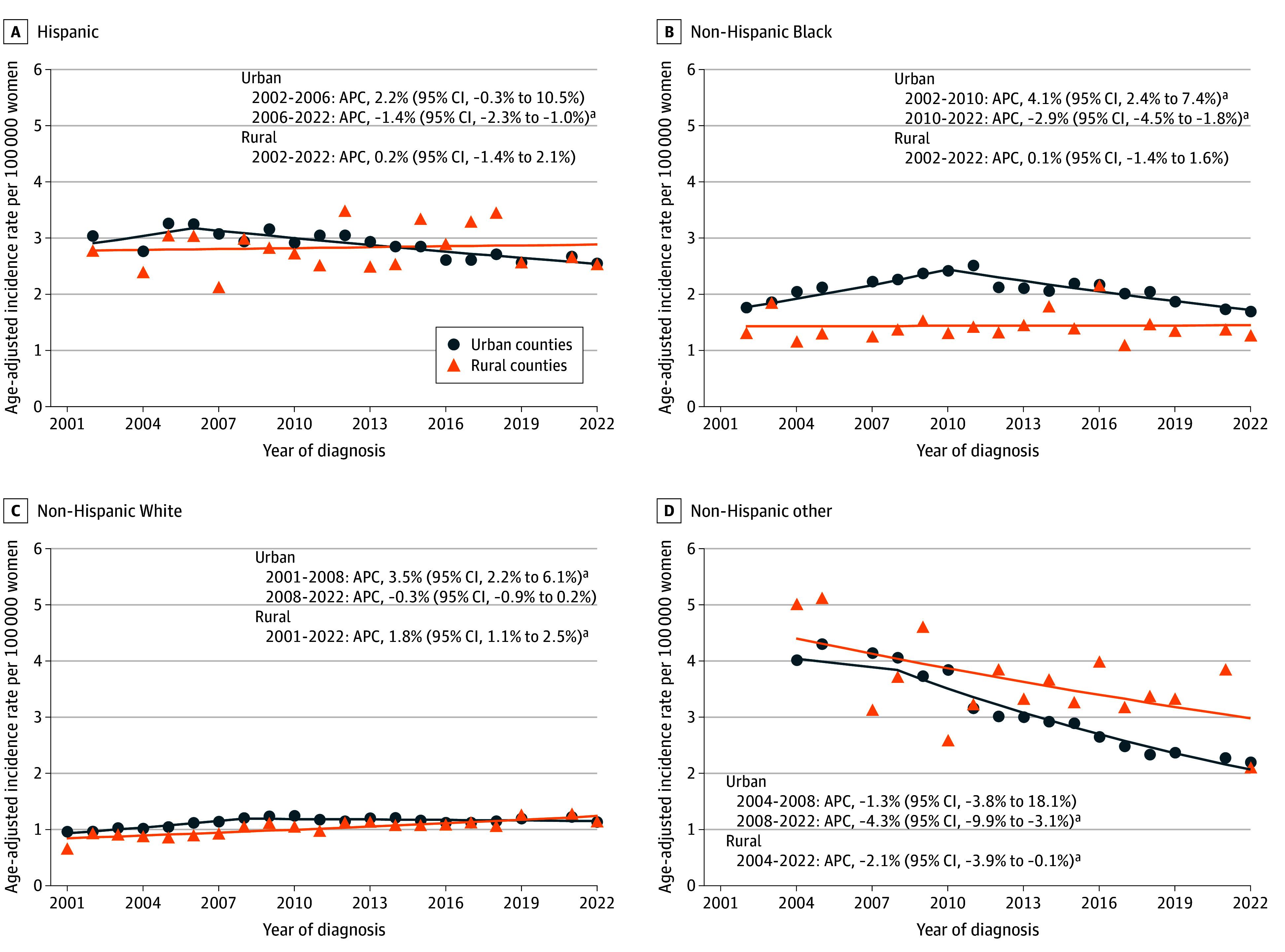
Scatterplots Showing Age-Adjusted Incidence of Hepatocellular Carcinoma Among Women in the US by Race and Ethnicity and Rurality, 2001-2022 The scatterplots display annual age-adjusted incidence rates of hepatocellular carcinoma in urban and rural counties between 2001 and 2022. Race and ethnicity were categorized as Hispanic, non-Hispanic Black, non-Hispanic White, and non-Hispanic other (American Indian or Alaska Native, Native Hawaiian or Pacific Islander, and unspecified race categories). Hepatocellular carcinoma cases were identified using the *International Classification of Diseases for Oncology, Third Edition* (site code C22.0; histology codes 8170-8175). Rurality was determined using the 2013 Rural-Urban Continuum Codes and categorized as urban counties (codes 1-3) and rural counties (codes 4-9). Incidence rates were age adjusted to the 2000 US standard population and expressed per 100 000 persons. Annual percentage changes (APCs) and 95% CIs were estimated using the Joinpoint Regression Program, version 5.4.0 (National Cancer Institute). Calendar segments correspond to statistically identified joinpoints. ^a^*P* < .05.

All results for trend analyses across all race and ethnicity groups are presented in the Table, Figure 2, and Figure 3. Among Hispanic and non-Hispanic Black men, no statistically significant change in trends was observed in rural areas (Hispanic: APC, 0.8% [95% CI, 0.0%-1.8%] from 2001 to 2022; non-Hispanic Black: APC, 0.1% [95% CI, −5.3% to 1.6%] from 2009 to 2022), while decreasing trends were observed in urban areas (Hispanic: APC, −2.2% [95% CI, −2.7% to −1.8%] from 2006 to 2022; non-Hispanic Black: APC, −2.7% [95% CI, −3.3% to −2.1%] from 2008 to 2022). Among non-Hispanic White men, the HCC incidence in rural counties increased by 1.1% (95% CI, 0.6%-1.6%) per year from 2007 to 2022, and the HCC incidence in urban counties decreased by 0.9% (95% CI, −1.3% to −0.6%) per year from 2008 to 2022 (Figure 2). Among women, an increase in HCC incidence of 1.8% (95% CI, 1.1%-2.5%) per year was observed among the non-Hispanic White population in rural counties from 2001 to 2022, whereas incidence rates in urban counties remained relatively stable after 2008 (APC, −0.3% [95% CI, −0.9% to 0.2%]) (Figure 3). Trends for the non-Hispanic American Indian or Alaska Native and non-Hispanic Asian or Pacific Islander populations are presented in eFigure 1 in Supplement 1.

Between 2001 and 2022, among men, HCC incidence increased more rapidly in rural areas than in urban counties across all stages. In particular, localized-stage HCC incidence increased by an APC of 4.0% (95% CI, 3.3%-5.2%) per year in rural counties compared with 1.5% (95% CI, 1.1%-1.8%) per year in urban counties, while regional-stage incidence increased by 2.6% (95% CI, 2.1%-3.2%) per year in rural counties and by 0.9% (95% CI, 0.3%-1.7%) per year in urban counties (Table; eFigure 2 in Supplement 1). Distant-stage incidence increased by 1.9% (95% CI, 0.6%-2.7%) per year in rural counties from 2007 to 2022, whereas incidence decreased by 0.6% (95% CI, −1.0% to −0.3%) per year in urban counties from 2008 to 2022. Similarly, among women, the incidence increased more rapidly in rural counties than in urban counties, with the most notable difference in the distant stage (2.0% [95% CI, 0.8%-3.3%] for rural counties vs 0.1% [95% CI, −0.2% to 0.6%] for urban counties) (Table; eFigure 3 in Supplement 1).

**Figure 4.**
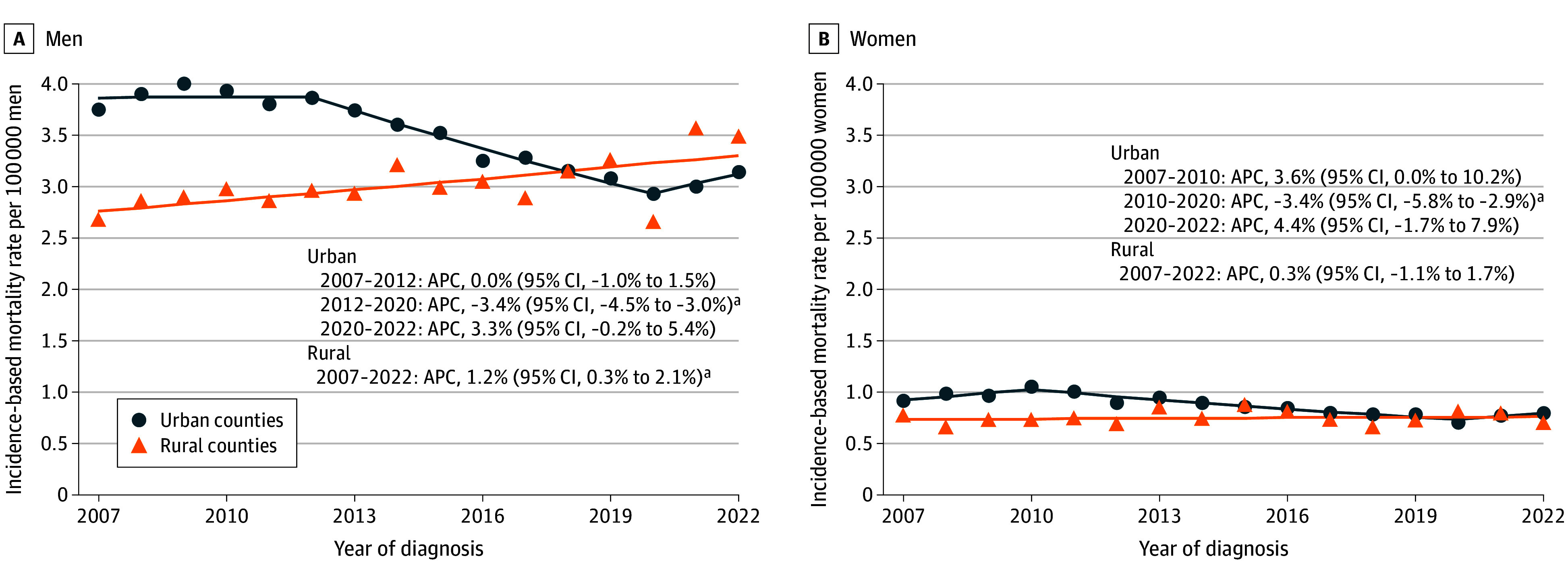
Scatterplots Showing Incidence-Based Mortality from Hepatocellular Carcinoma by Sex and Rurality in the US, 2007-2022 The scatterplots display annual age-adjusted incidence-based mortality rates from hepatocellular carcinoma in urban and rural counties between 2007 and 2022. Incidence-based mortality included liver cancer deaths among individuals who previously received a diagnosis of hepatocellular carcinoma, based on the Surveillance, Epidemiology, and End Results (SEER)–21 incidence-based mortality file, which links SEER-21 incidence data with death certificate records. Rurality was determined using the 2013 Rural-Urban Continuum Codes and categorized as urban counties (codes 1-3) and rural counties (codes 4-9). Rates were age adjusted to the 2000 US standard population and expressed per 100 000 persons. Annual percentage changes (APCs) and average annual percentage changes with 95% CIs were estimated using the Joinpoint Regression Program, version 5.4.0 (National Cancer Institute). ^a^*P* < .05.

From 2007 to 2022, among men, incidence-based mortality rates were 2.7 (95% CI, 2.3-3.0) per 100 000 people in rural counties and 3.8 (95% CI, 3.6-3.9) per 100 000 people in urban counties (Table and Figure 4). However, incidence-based mortality rates increased by 1.2% (95% CI, 0.3%-2.1%) per year in rural counties and decreased by an APC of 1.4% (95% CI, −1.7% to −1.1%) per year in urban counties. Among women, incidence-based mortality rates were 0.8 (95% CI, 0.6-0.9) per 100 000 people in rural counties and 0.9 (95% CI, 0.8-1.0) per 100 000 people in urban counties. Incidence-based mortality rates remained stable in rural areas (0.3% [95% CI, −1.1% to 1.7%] per year), while the rates decreased by 1.0% (95% CI, −1.7% to −0.4%) per year on average in urban areas.

For incidence-based mortality trends, rates among non-Hispanic White men in rural areas increased (1.2% [95% CI, 0.5%-2.0%] per year), and among non-Hispanic White women in rural areas, rates tended to increase (0.6% [95% CI, −0.7% to 2.0%] per year) (Table). However, incidence-based mortality rates decreased in urban areas (non-Hispanic White men: −0.8% [95% CI, −1.2% to −0.4%] per year; non-Hispanic White women: −1.7% [95% CI, −2.7% to −0.8%] per year). Incidence-based mortality rates among Hispanic men (0.6% [95% CI, −1.8% to 3.4%] per year) and Black men (2.5% [95% CI, −0.4% to 4.6%] per year) remained stable in rural areas and decreased in urban areas (Hispanic men: −2.8% [95% CI, −3.4% to −2.1%] per year; non-Hispanic Black men: −2.0% [95% CI, −3.3% to −0.8%] per year).

## Discussion

Our findings reveal rural-urban disparities in HCC incidence and incidence-based mortality trends, particularly since 2008. Recent HCC incidence has continued to increase in rural counties, particularly among the non-Hispanic White population. In contrast, HCC incidence has decreased or plateaued in urban counties. These findings suggest that improvements in HCC prevention and management have not been uniformly distributed across geographic populations.

The findings of the study support the initial hypothesis that changing key risk factors for HCC affects rural and urban regions disproportionately. The inflection in HCC incidence trends may reflect the long-term changes in viral hepatitis–related liver disease, such as decreased transmission and advances in prevention, screening, and treatment.^2^ Because of the lengthy latency between chronic viral hepatitis infection and the development of HCC, these trends probably reflect the delayed effect of earlier shifts in viral hepatitis epidemiology, while MASH, obesity, diabetes, and ALD are now emerging as leading contributors to HCC risk^8,9,10,11,12^ and are more prevalent in rural populations.^13,14^ For example, national surveillance data show that obesity affects approximately 34% of adults in rural areas compared with approximately 29% in urban areas,^15^ and diabetes prevalence is similarly higher in rural populations at approximately 14% vs 11% in urban areas.^16^ Alcohol use disorder and heavy drinking patterns are more prevalent in rural communities, contributing to a disproportionate burden of ALD. Alcohol-induced death rates in rural areas are 18% to 23% higher than in urban populations, and recent studies report higher ALD mortality among rural residents, including 9.0 vs 7.8 deaths per 100 000 people among rural vs urban men.^17,18^

Our study found that the pronounced increase in HCC incidence and mortality was observed among non-Hispanic White populations in rural counties. The shifting etiologic profile from viral hepatitis, which disproportionately affected certain racial and ethnic minority populations and urban settings,^19^ to MASH and ALD^20^ may partly explain this finding. These conditions are more prevalent in rural populations and among non-Hispanic White individuals.^21^ In addition, a prior study reported that ALD mortality increased by 4.4% per year among non-Hispanic White populations in rural counties, whereas no similar increase was observed among other racial and ethnic populations except non-Hispanic American Indian or Alaska Native populations.^13^

This study has revealed an increase of 2% per year in the incidence of advanced-stage HCC in rural counties. Considering that residents of rural areas face significant barriers to accessing both preventive and specialized care, such as a lack of hepatologists, longer travel distances to tertiary centers, and lower screening rates for cirrhosis and HCC,^22,23,24,25^ these barriers may delay diagnosis and reduce access to timely surveillance for individuals at high risk. Our results highlight the need for targeted prevention and improved access to care in rural counties.

Our findings also show that the historically higher incidence of HCC in urban counties has narrowed in recent years. The gap in liver cancer deaths among patients with HCC between rural and urban counties has closed; among men, rural counties now show higher incidence-based mortality rates compared with urban areas. The increasing incidence-based mortality rates in rural regions may be explained by the increasing incidence of advanced-stage HCC, as we reported, and the lower survival rates in these areas. Several studies have documented reduced access to transplants and locoregional therapies in rural areas and poorer survival among patients with HCC in rural areas compared with urban settings.^24,26,27^

Consistent with prior studies, sex differences still exist in HCC incidence and mortality. Men showed higher incidence rates than women in both rural and urban areas.^28,29^ This gap likely results from greater exposure to major risk factors, such as alcohol consumption, chronic viral hepatitis, and MASH, along with possible biological factors, such as androgen-related promotion of hepatocarcinogenesis.^30^

### Limitations

This study has several limitations. First, although we excluded 2020 due to potential associations of COVID-19 with incidence of HCC, these associations may have continued into 2021 and 2022. COVID-19 may also have been associated with incidence-based mortality rates, particularly for the years 2020 to 2022. Second, while we used the 2024 submission, there could be delays in reporting that particularly affect incidence rates for 2022 and incidence-based mortality rates in recent years. Third, we observed decreasing trends in the incidence of unknown or unstaged cancers. As a result, the increasing availability of stage information over time may have influenced trends for localized, regional, and distant stages.

## Conclusions

This cohort study provides comprehensive national evidence of rural-urban disparities in HCC incidence and mortality trends. These findings highlight the need for targeted strategies to improve prevention, early detection, and access to specialized care for liver disease in rural communities.

## Notes

Supplement 1. **eFigure 1. **Non-Hispanic Other Race-Specific Hepatocellular Carcinoma Incidence Trends by Rurality, United States, 2001–2022 **eFigure 2. **Age-Adjusted Incidence of Hepatocellular Carcinoma by Rurality and Stage at Diagnosis Among Men, United States, 2001–2022 **eFigure 3. **Age-Adjusted Incidence of Hepatocellular Carcinoma by Rurality and Stage at Diagnosis Among Women, United States, 2001–2022

Supplement 2.
Data Sharing Statement
