# Supplementary material for: Rural-Urban Disparities in Hepatocellular Carcinoma Incidence and Mortality
Source: JAMA Netw Open. 2026 May 13;9(5):e2612323. doi: 10.1001/jamanetworkopen.2026.12323 (PMC13173385; doi:10.1001/jamanetworkopen.2026.12323)
Supplement: Supplement 1. — eFigure 1. Non-Hispanic Other Race-Specific Hepatocellular Carcinoma Incidence Trends by Rurality, United States, 2001–2022 eFigure 2. Age-Adjusted Incidence of Hepatocellular Carcinoma by Rurality and Stage at Diagnosis Among Men, United States, 2001–2022 eFigure 3. Age-Adjusted Incidence of Hepatocellular Carcinoma by Rurality and Stage at Diagnosis Among Women, United States, 2001–2022 [file jamanetwopen-e2612323-s001.pdf]

Supplementary Online Content

Damgacioglu H, Uygun G, Deshmukh AA, Kaseb AO, Noonan AM, Demir T. Rural-urban disparities in hepatocellular carcinoma incidence and mortality in the US, 2001-2022. *JAMA Netw Open.* 2026;9(5):e2612323. doi:10.1001/jamanetworkopen.2026.12323

eFigure 1. Non-Hispanic Other Race-Specific Hepatocellular Carcinoma Incidence Trends by Rurality, United States, 2001–2022

eFigure 2. Age-Adjusted Incidence of Hepatocellular Carcinoma by Rurality and Stage at Diagnosis Among Men, United States, 2001–2022

eFigure 3. Age-Adjusted Incidence of Hepatocellular Carcinoma by Rurality and Stage at Diagnosis Among Women, United States, 2001–2022

This supplementary material has been provided by the authors to give readers additional information about their work.

© 2026 Damgacioglu H et al. *JAMA Network Open.*

eFigure 1. Non-Hispanic Other Race-Specific Hepatocellular Carcinoma Incidence Trends by Rurality, United States, 2001–2022
(A) Non-Hispanic American Indian or Alaska Native (AIAN) men; (B) Non-Hispanic Asian or Pacific Islander (NHAPI) men; (C) AIAN women; (D) NHAPI women.

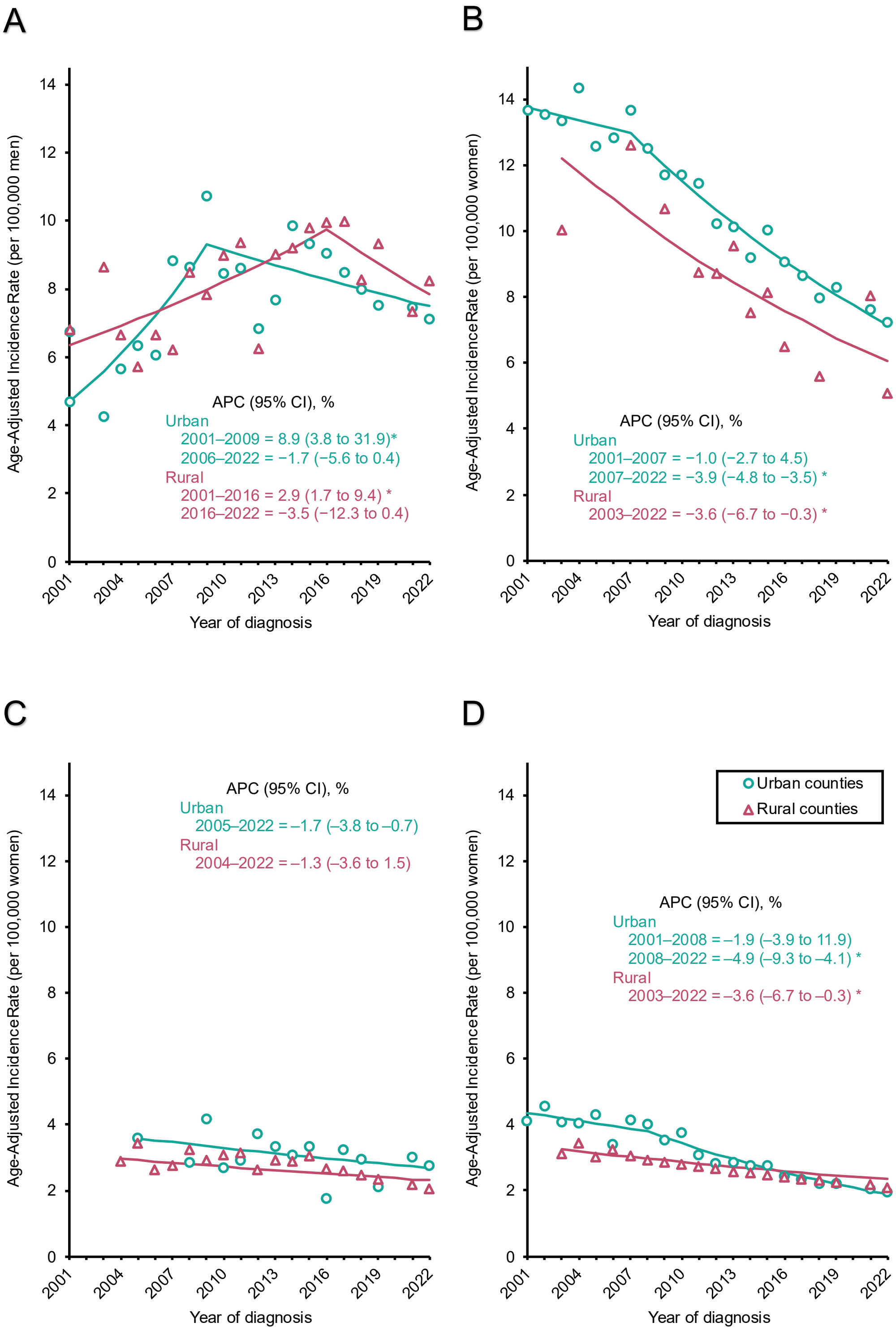

Abbreviation: APC, annual percent change.

Hepatocellular carcinoma cases were identified using the International Classification of Diseases for Oncology, Third Edition (site code C22.0; histology codes 8170–8175).

Rurality was determined using the 2013 Rural–Urban Continuum Codes and categorized as urban counties (codes 1–3) and rural counties (codes 4–9).

Incidence rates were age-adjusted to the 2000 US standard population and expressed per 100 000 persons.

Circles represent observed incidence rates for urban counties and triangles represent observed incidence rates for rural counties. Solid lines indicate modeled incidence trends estimated using Joinpoint regression.

Annual percent changes (APCs) and 95% confidence intervals (CIs) were estimated using the Joinpoint Regression Program (version 5.4.0; National Cancer Institute); * indicates P < .05.

© 2026 Damgacioglu H et al. *JAMA Network Open.*

eFigure 2. Age-Adjusted Incidence of Hepatocellular Carcinoma by Rurality and Stage at Diagnosis Among Men, United States, 2001–2022

(A) Localized; (B) Regional; (C) Distant; (D) Unknown or unstaged.

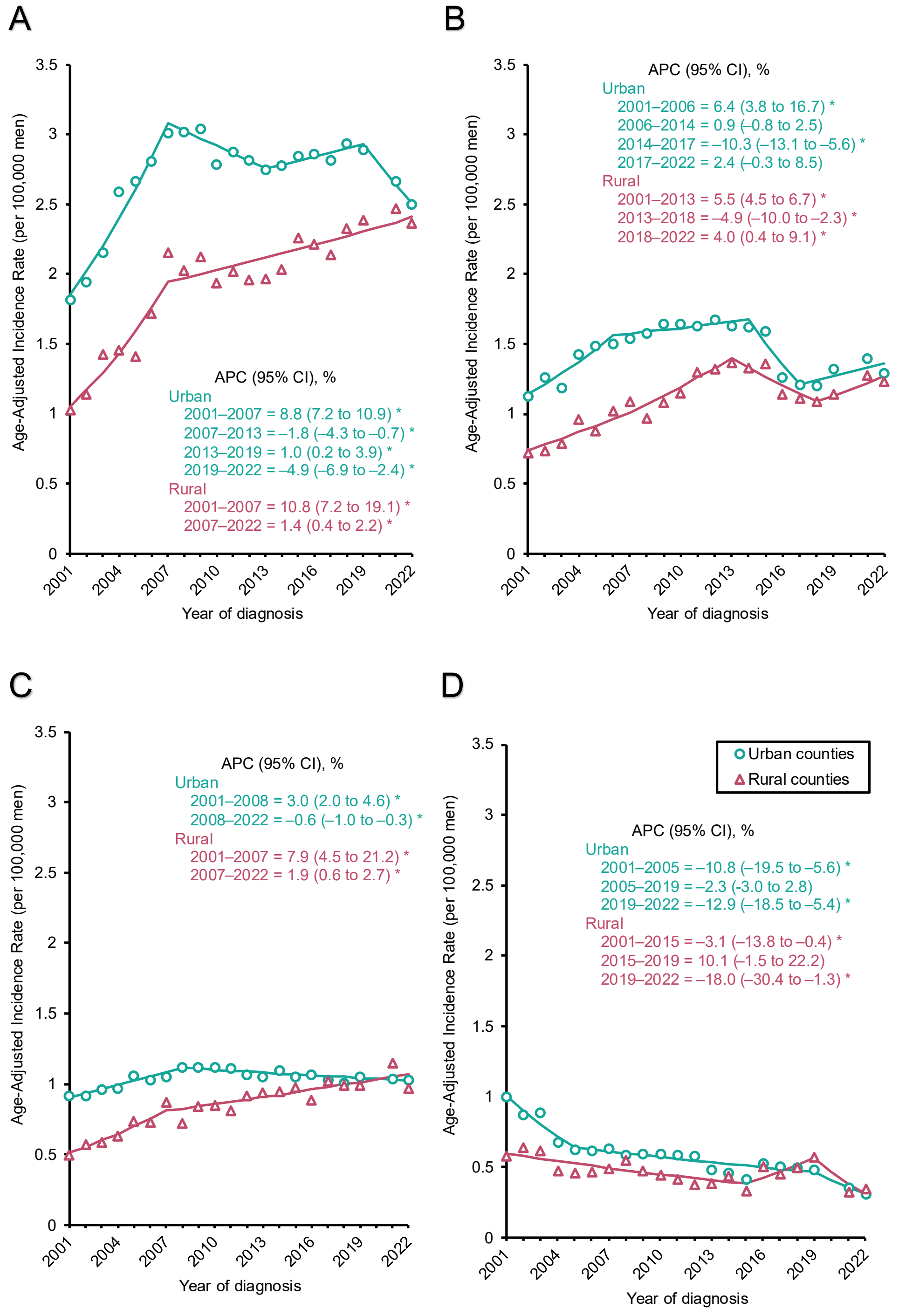

Abbreviation: APC, annual percent change.

Hepatocellular carcinoma cases were identified using the International Classification of Diseases for Oncology, Third Edition (site code C22.0; histology codes 8170–8175).

Stage at diagnosis was classified using the SEER Summary Stage system.

Rurality was determined using the 2013 Rural–Urban Continuum Codes and categorized as urban counties (codes 1–3) and rural counties (codes 4–9).

Incidence rates were age-adjusted to the 2000 US standard population and expressed per 100 000 persons.

Circles represent observed incidence rates for urban counties and triangles represent observed incidence rates for rural counties. Solid lines indicate modeled incidence trends estimated using Joinpoint regression.

Annual percent changes (APCs) and 95% confidence intervals (CIs) were estimated using the Joinpoint Regression Program (version 5.4.0; National Cancer Institute). Calendar segments correspond to statistically identified joinpoints; * indicates P < .05.

© 2026 Damgacioglu H et al. *JAMA Network Open.*

eFigure 3. Age-Adjusted Incidence of Hepatocellular Carcinoma by Rurality and Stage at Diagnosis Among Women, United States, 2001–2022
(A) Localized; (B) Regional; (C) Distant; (D) Unknown or unstaged.

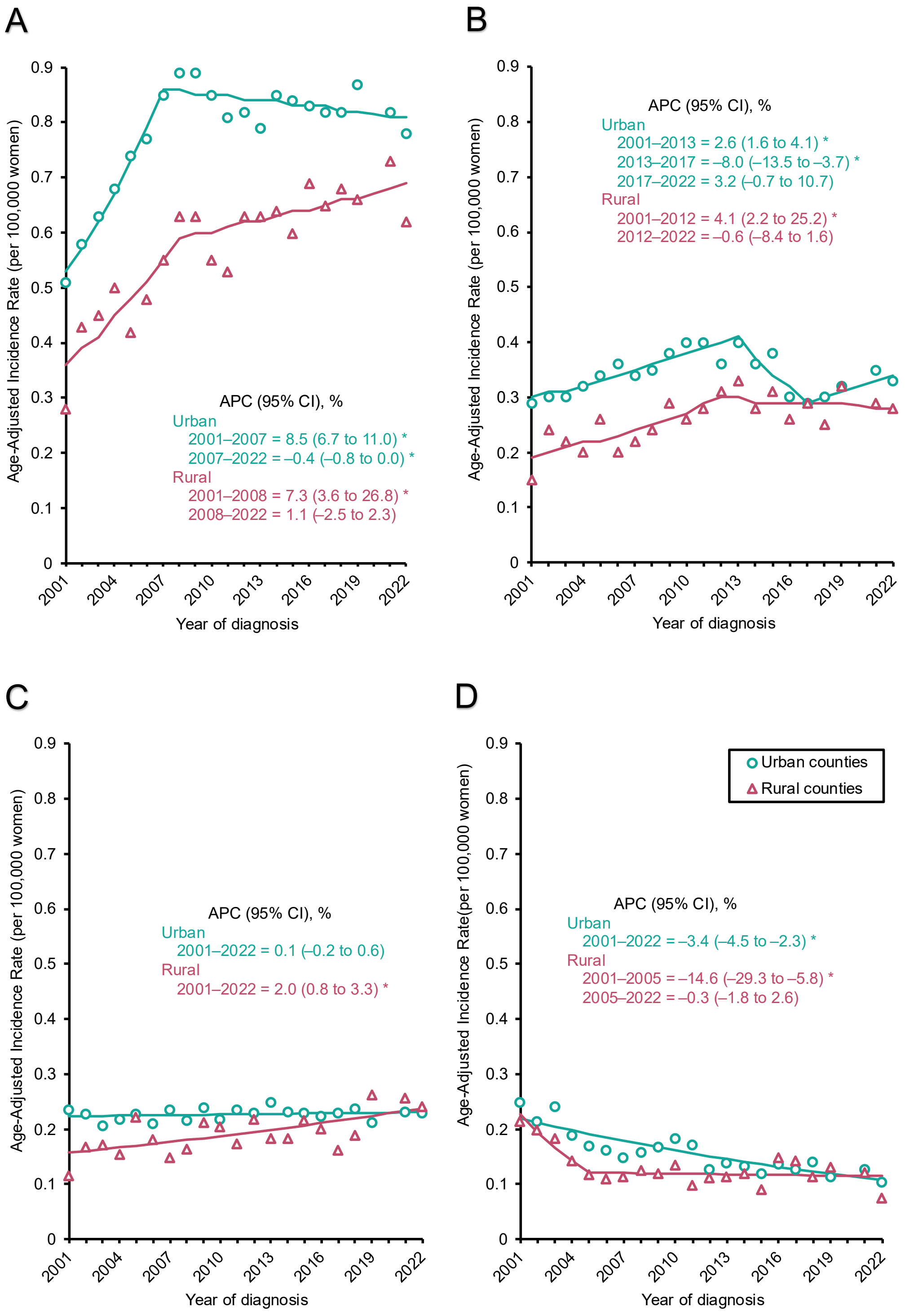

Abbreviation: APC, annual percent change. Legend text identical to eFigure 2, except analyses were performed among women.

© 2026 Damgacioglu H et al. *JAMA Network Open.*
