# Supplementary material for: Rural-Urban Disparities in Hepatocellular Carcinoma Incidence and Mortality
Source: JAMA Netw Open. 2026 May 13;9(5):e2612323. doi: 10.1001/jamanetworkopen.2026.12323 (PMC13173385; doi:10.1001/jamanetworkopen.2026.12323)
Supplement: Supplement 2. — Data Sharing Statement [file jamanetwopen-e2612323-s002.pdf]

Data Sharing Statement

Damgacioglu. Rural-Urban Disparities in Hepatocellular Carcinoma Incidence and Mortality in the US. *JAMA Netw Open*. Published May 13, 2026.
doi:10.1001/jamanetworkopen.2026.12323

Data
Data available: No

Additional Information
Explanation for why data not available: The data used in the study is publicly available
